# Supplementary material for: Simple Process for Flexible Light-Extracting QD Film and White OLED
Source: Micromachines (Basel). 2025 Nov 30;16(12):1367. doi: 10.3390/mi16121367 (PMC12734796; doi:10.3390/mi16121367)
Supplement: Supplementary file 1 [file micromachines-16-01367-s001.zip › micromachines-3983644-supplementary.pdf]

(Supporting Information)

Simple Process for Flexible Light-Extracting QD Film and White OLED

Eun Jeong Bae [1,2], Tae Jeong Hwang [2], Geun Su Choi [1,2], Yong-Min Lee [3], Byeong-Kwon Ju [1], Young Wook Park [2,4,*] and Dong-Hyun Baek [4,*]

[1] Display and Nanosensor Laboratory, Department of Electrical Engineering, Korea University, 145, Anam-ro, Seongbuk-gu, Seoul 02841, Republic of Korea; baeejng@korea.ac.kr (E.J.B.); bkju@korea.ac.kr (B.-K.J.)

[2] Nano and Organic-Electronics Laboratory, Department of Display and Semiconductor Engineering, Sunmoon University, Asan 31460, Chungcheongnam-do, Republic of Korea; zeratull1234@sunmoon.ac.kr

[3] Research Center for Nano-Bio Science, Sunmoon University, Asan 31460, Chungcheongnam-do, Republic of Korea; ymlee@sunmoon.ac.kr

[4] Center for Next Generation Semiconductor Technology, Department of Display and Semiconductor Engineering, Sunmoon University, Asan 31460, Chungcheongnam-do, Republic of Korea

* Correspondence: zerook@sunmoon.ac.kr (Y.W.P.); dhbaek@sunmoon.ac.kr (D.-H.B.)

1. EQE calculation using viewing profile

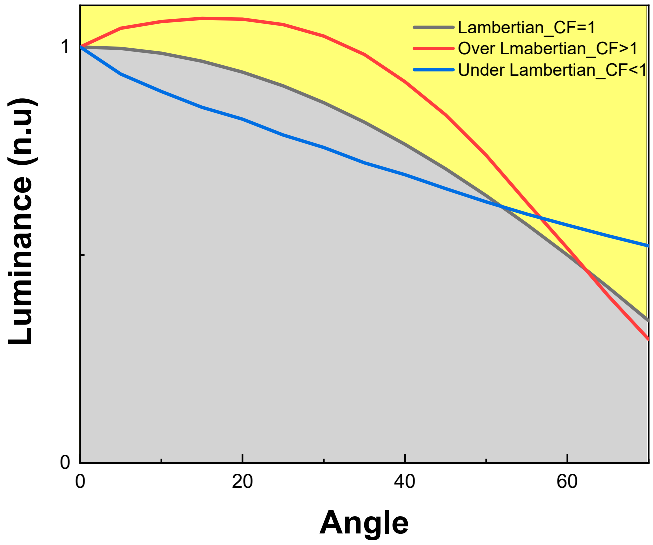

Figure S1. Schematic illustration of angular emission profiles compared with the Lambertian distribution.

The EQE must be calculated using the total luminous flux, but precise measurement is difficult at the laboratory scale because it requires expensive large-scale 3D automated measurement systems or integrating measurement systems using goniometers or integrating spheres. Therefore, a simple calculation method using the field-of-view angle profile is widely used in this research, and it consists of the following procedure.

1) Measure the EL characteristics with perpendicular emission (incident angle = 0˚).
2) Calculation of the total luminous flux assuming the light source has Lambertian emission profile, and calculate the 'EQE$_{\text{assuming Lambertian}}$'
3) Measure the viewing angle profile
4) Calculate the conversion factor compared to the Lambertian light source
5) Apply conversion factor to the previously calculated 'EQE$_{\text{assuming Lambertian}}$'.
6) EQE = conversion factor * 'EQE$_{\text{assuming Lambertian}}$'.

To quantitatively evaluate the effect of angular emission characteristics on light extraction efficiency, a conversion factor (CF) was calculated based on the ideal Lambertian profile. First, the total luminous flux was estimated by assuming a Lambertian emission distribution ($I = I_0\cos\theta$) from the front luminance at 0° incidence angle, and the 'EQE Lambertian' was calculated accordingly. Then, the actual angular emission profile of each sample was measured, and the ratio of the integrated intensity compared to the Lambertian distribution was used to determine the CF. When the CF is greater than 1, the emission is more concentrated laterally (Over Lambertian), whereas CF values less than 1 indicate a broader emission profile with relatively weaker forward emission (Under Lambertian). The corrected EQE was obtained using the following equation:

$$EQE = CF \times EQE_{Lambertian}$$

Although the EQE should be calculated using total luminous flux, it is hard to measure precisely on a lab-scale since it requires a large scale and highly expensive 3D automation measurement system or integral measurement system using a goniometer or integral spheres. So, the simple calculation using viewing angle profile is widely used and adopted in this work. It consists of the following procedures.
